# Transfusion therapy in paediatric trauma patients: a review of the literature

**DOI:** 10.1186/s13049-015-0097-z

**Published:** 2015-02-15

**Authors:** Kristin Brønnum Nystrup, Jakob Stensballe, Morten Bøttger, Pär I Johansson, Sisse R Ostrowski

**Affiliations:** Section for Transfusion Medicine, Capital Region Blood Bank, Rigshospitalet, Copenhagen University Hospital, Blegdamsvej 9, Copenhagen, DK-2100 Denmark; Department of Paediatrics, Næstved Hospital, Ringstedgade 61, Næstved, DK-4700 Denmark; Department of Anaesthesiology, Centre of Head and Orthopaedics, Rigshospitalet, Copenhagen University Hospital, Blegdamsvej 9, Copenhagen, DK-2100 Denmark; Department of Surgery, Division of Acute Care Surgery, Centre for Translational Injury Research (CeTIR), University of Texas Medical School at Houston, Houston, TX USA

**Keywords:** Trauma, Paediatric, Transfusion, Coagulopathy, Transfusion adverse effects, Volume resuscitation

## Abstract

Haemorrhage is a leading cause of death in paediatric trauma patients. Predefined massive transfusion protocols (MTP) have the potential to significantly reduce mortality by treating haemorrhagic shock and coagulopathy, in adhering to the principles of haemostatic resuscitation with rapid administration of balanced ratios of packed red blood cells (RBC), fresh frozen plasma (FFP) and platelets (PLT).

Because of their substantial physiological reserve, initial vital signs may not be good predictors of early haemorrhage in paediatric patients. Determining the triggers for MTP activation in paediatric trauma patients is challenging, and the optimal blood product ratio that will increase survival in massively bleeding paediatric trauma patients has yet to be determined. To date, only a few small descriptive studies and case reports have investigated the use of predefined MTP in paediatric trauma patients.

MTP with increased FFP or PLT to RBC ratios combined with viscoelastic haemostatic assay (VHA) guided haemostatic resuscitation have not yet been tested in paediatric populations but based on results from adult trauma patients, this therapeutic approach seems promising.

Considering the high prevalence of early coagulopathy in paediatric trauma patients, immediate identification and implementation of VHA-directed treatment of traumatic coagulopathy could ensure faster haemostasis and thereby, potentially, reduce bleeding as well as the total transfusion requirements and further improve outcome in paediatric trauma patients. Prospective randomized trials investigating this therapeutic approach in paediatric trauma patients are highly warranted.

## Introduction

Globally, injuries account for an estimated 950,000 deaths annually in children less than 18 years of age and in high-income countries, injuries cause nearly 40% of all child deaths [1]. Leading causes of death in paediatric trauma patients include traumatic brain injury (TBI) and haemorrhage [2,3].

Coagulopathy is present in about one third of adult trauma patients on emergency department (ED) arrival [4,5]. Traumatic coagulopathy is at least as prevalent in paediatric trauma patients and is, similar to adults, associated with increased morbidity and mortality [3,6-8]. Massive transfusion protocols (MTP) are designed to provide the right amount and balance of blood products, mimicking whole blood, to critically injured patients in order to prevent and treat haemorrhagic shock and coagulopathy [9,10]. MTPs are based on the recently developed concept of damage control resuscitation (DCR), which advocates early blood component therapy together with minimal crystalloid use directed towards hypotensive resuscitation whilst avoiding haemodilution, combined with rapid surgical control [11,12].

An intricate part of the DCR concept is a balanced transfusion strategy with packed red blood cells (RBC), fresh frozen plasma (FFP) and platelets (PLT) in a 1:1:1 unit ratio with the appropriate use of coagulation factors such as fibrinogen-containing products, prothrombin complex concentrate and recombinant FVIIa, alternatively fresh whole blood where available. This transfusion strategy is termed haemostatic resuscitation (HR) [11,12]. The purpose of the overall DCR concept is to alleviate the complications of hypoperfusion, acidosis, hypothermia and coagulopathy that often accompany substantial haemorrhage in patients with severe traumatic injuries [11,13,14].

Early administration of predefined balanced ratios of RBC, FFP and PLT have been shown to be associated with improvements in patient outcome in adult trauma and non-trauma patients [15-18], though the optimal ratio of PLT and FFP to RBC is currently being investigated in a randomized controlled trial [19]. As in adults, the optimal ratio for blood product administration in paediatric trauma patients in need of massive transfusion is unknown [20-22]. There is an urgent need for evidence based guidelines on massive transfusion therapy for this population [23,24]. The purpose of this review is to summarise the current evidence regarding transfusion therapy in massively bleeding paediatric trauma patients.

### Special considerations in the paediatric trauma patient

Massive bleeding has historically been defined as the loss of one or more circulating blood volumes and in paediatric patients, all estimates of blood volume, volume loss and volume replacement are based on weight with children over the age of 3 months having an estimated blood volume of 70 ml/kg, and younger infants having an estimated 90 ml/kg [23-25].

The clinical signs and symptoms of hypovolaemia in children may vary from adults because of their substantial physiological reserve, and initial vital signs may not be good predictors of early haemorrhage. Children are able to maintain a normal blood pressure until a loss of more than 20% of their blood volume [2,20,25]. A narrow pulse pressure may be a more sensitive sign of hypovolaemia than tachycardia or systolic hypotension, and metabolic acidosis secondary to hypoperfusion and decreased urine output are additional indicators of hypovolaemia [25]. Like adults, large amounts of blood may be lost internally secondary to long bone fractures, retroperitoneal or abdominal trauma and, unique to children, substantial bleeding may occur due to closed head trauma [25].

Clinical monitoring should focus on perceived tissue oxygenation with continuous measurements of heart rate, pulse oximetry, arterial blood pressure and in massively bleeding patients, invasive monitoring should include repeated measurements of lactate and mixed venous oxygen saturation [21]. Children are at least as capable as adults in their ability to compensate for lower haemoglobin concentrations with increased oxygen extraction and cardiac output [26], despite their limited myocardial compliance [21]. However, compared to adults, children have increased energy requirements resulting in higher tissue oxygen utilization [21], and a transient increase in brain tissue oxygen tension after RBC transfusion has been found [27], emphasizing the utility of RBC for increasing oxygen delivery to hypoxic tissues. In this study, the majority (70%) of the transfused RBC had been stored ≤ 14 days.

A specific level of blood loss or anaemia that triggers RBC transfusion has not yet been defined in paediatric patients [23,24]. A study done on haemodynamically stable, critically ill paediatric patients, the TRIPICU trial, showed no adverse effects when comparing a restrictive transfusion strategy initiated at a haemoglobin level of 7 g/dl to a transfusion threshold of 9.5 g/dl [28]. A specific anaemia threshold would be difficult to use as a trigger for MTP activation in the case of massive haemorrhage, because haemoglobin and haematocrit levels might not reveal significant anaemia until the patient had been volume resuscitated, making a precise estimate of lost blood volume difficult to ascertain based on these laboratory tests [23,25].

### The paediatric haemostatic system – mature from 6 months of age

The haemostatic system is incompletely developed at birth and matures throughout infancy. Both full-term and preterm neonates are born with low levels of most procoagulant proteins including all the contact activation factors and vitamin K-dependent factors [29]. Similarly, levels of the major anticoagulant proteins (tissue factor pathway inhibitor, antithrombin, protein C) are low at birth. The concentrations of these proteins remain low compared to adults until approximately 6 months of age [29-31]. Neonatal platelet counts do not differ from that in adults, but neonatal fibrinogen is qualitatively dysfunctional existing in a foetal form until approximately 6 months to 1 year of age. Furthermore, plasminogen is both quantitatively and qualitatively different from that in adults until 6 months of age. This leads to reduced plasmin generation and fibrinolytic activity of infants [29]. The differences compared to adults are primarily quantitative [30], ensuring that neonates and infants have an overall balanced and efficient haemostasis, unless disrupted by critical illness [29]. Due to the reduced concentration of procoagulant proteins, it might be assumed that young infants are at higher risk of bleeding than adults, since this will prolong the prothrombin time (PT) and activated partial thromboplastin time (APTT). Relying on transfusion thresholds based on these standard plasma based coagulation tests could, however, lead to over transfusion of blood products such as FFP [32,33]. Functional viscoelastic assays analyzing whole blood may provide more accurate information since they evaluate the entire haemostatic process [34], as described later.

### Physiological predictors of adverse outcome

A high Injury Severity Score (ISS), shock [7,35] and high base deficit [7,36] on admission are all independent predictors of increased mortality in the general paediatric trauma population.

Severe TBI is the leading cause of death in children over the age of 1 year, and since hypoxia and hypotension are major causes of secondary brain lesions that worsen outcome, initial resuscitation efforts should aim at minimizing the extent and duration of these conditions [35]. Several investigators have confirmed that low Glasgow Coma Scale (GCS) score on admission is an independent predictor of mortality in paediatric patients with TBI [7,22,35].

Patregnani et al. found that coagulopathy (international normalized ratio (INR) ≥ 1,5) on admission was common and associated with increased mortality in children with traumatic injuries, independent of the ISS [7]. The same association was shown by other investigators, who also found that the incidence of coagulopathy in paediatric trauma patients increased with higher ISS and in patients with TBI [6,37,38]. Vavilala et al. found that the presence of coagulopathy (defined by increased fibrin degradation products) independently predicted poor outcome in children with isolated head injury [39]. Several mechanisms have been proposed to explain the coagulation abnormalities associated with TBI, which show a combination of both hypocoagulable and hypercoagulable states [40]. It has also been hypothesized that the trauma causes local release of tissue factor from the injured neurons, which is associated with activation of the protein C pathway, thus triggering the release of anticoagulant mediators [34,41].

Borgman et al. found both admission base deficit < 8 and INR > 1.8 to be independently associated with mortality, and proposed that a score based on these predictors of adverse outcome (“BIG” score: base deficit + [2.5 × INR] + [15 – GCS score]) may more accurately predict mortality in paediatric trauma patients than scoring systems currently in use [42].

Measurements of admission base deficit are frequently used as markers of tissue hypoperfusion and shock. The prognostic value of lactate levels has not yet been defined in paediatric patients. Several investigators suggest using shock index (defined as heart rate/systolic blood pressure) as an indicator of tissue perfusion as it reflects both vascular and myocardial dysfunction [43,44].

The presence of hypovolaemic shock in paediatric trauma patients with coinciding coagulopathy correlates well with data from adult trauma populations, where drivers of trauma-induced coagulopathy include acidosis as a result of hypovolaemic shock and consumption of coagulation factors secondary to local activation of the coagulation system after severe traumatic injury [34,45]. A distinct mechanism of trauma-induced coagulopathy involving activation of the anticoagulant protein C pathway, caused by tissue hypoperfusion [41,45] has also been described, and recently high endogenous catecholamine levels and endothelial damage have also been proposed to promote coagulopathy in trauma [46-48].

### Massive transfusion protocols in paediatric trauma patients

Quick instigation of a balanced transfusion strategy with RBC, FFP and PLT in a 1:1:1 unit ratio combined with early, repeated coagulation monitoring and goal-directed transfusion therapy, are intricate parts of the HR concept as described above [10-12]. A restrictive use of crystalloids to decrease coagulopathy induced by haemodilution is also emphasized [10,11]. The adverse effects of haemodilution in children were described by Hussmann et al., who found that increased prehospital crystalloid volume replacement was associated with increased transfusion requirements, adversely affected coagulation (defined by a prolonged PT) and a tendency towards increased mortality and multi organ failure (MOF) rates [49].

Resuscitation with appropriate volumes and blood products is critical because both anaemia and hypovolemia decrease oxygen delivery to the tissues [21,25]. Besides containing fibrinogen, which is essential for clot formation, FFP is an excellent volume expander. So is RBC, and patients that require massive transfusion should therefore be given RBC and FFP as volume therapy to treat their hypovolemia causing hypoperfusion with ensuing oxygen debt and coagulation factor depletion, rather than crystalloids [16].

Studies in adult populations have shown that an increased ratio of PLT and FFP to RBC show an association with improved survival in massively bleeding trauma and non-trauma patients [15,16,50,51]. Nosanov et al. did not find the same association between increased FFP or PLT to RBC ratios and decreased mortality in massively transfused paediatric trauma patients [22]. This group defined massive transfusion as more than 50% of total blood volume lost within 24 hours. However, this 24 hour based definition of massive transfusion does not differentiate between patients with different bleeding dynamics in the hours after the trauma (massive initial bleeding versus later moderate, prolonged bleeding). Moreover, the study population was small (105 patients), low and heterogenous in their component ratios and all fatalities within the first 24 hours were excluded from the study, making the results susceptible to indication bias.

Use of warm fresh whole blood used in combat settings for adult patients with traumatic injuries have shown to be independently associated with improved 30-day survival [52]. Manno et al. conducted a small randomized controlled study of 161 children undergoing open heart surgery with cardiopulmonary bypass, meeting postoperative transfusion requirements with either 24–48 hour old whole blood stored at 4 degrees Celcius or reconstituted whole blood (RBC/FFP/PLT). They found that there was significantly less postoperative blood loss in the group receiving fresh whole blood, and ascribed this to better functioning platelets [53]. Recently, refrigerated whole blood treated with pathogen reduction technology maintained in vitro haemostatic function for at least 10–14 days if stored appropriately at 4 degrees Celcius [54]. Based on these data, whole blood could potentially be used as an alternative to reconstituted whole blood in trauma settings for haemostatic resuscitation, in centres that are able to handle and store it correctly. More investigation into the utility of whole blood in the emergency setting for paediatric resuscitation is warranted.

At present, only two single-centre studies and few case reports have documented their experiences with the use of a predefined MTP in paediatric patients (Table 1). Hendrickson et al. conducted a study comparing the outcomes of 53 patients receiving a predefined MTP with 49 historic controls. This MTP contained weight-based blood product “packages” with fixed balanced ratios of blood products. The intended ratio of FFP:RBC was 1:1, and every other package also contained apheresis platelets or cryoprecipitate units. Only 50 percent of the patients in the MTP group required massive transfusion (defined as > 70 ml/kg total blood products transfused). Massively transfused patients received twice the ratio of FFP:RBC as the historic controls (1:1.8 compared to 1:3.6). The group did not find a statistically significant reduction in mortality (38% vs. 23%, p = 0.35) after MTP implementation when taking injury severity and coagulopathy (Table 1) into account in the multivariate analysis. Importantly, the majority of patients in both the pre-MTP and MTP group (80% vs. 72%) were found to have at least one abnormal coagulation value at presentation to the ED [3].

**Table 1:** **Studies evaluating the effect of a massive transfusion protocol in paediatric trauma patients**

**Author**	**No.**	**Type of study**	**FFP:RBC ratio high vs. low**	**PLT:RBC ratio high vs. low**	**Mortality high vs. low**	**Comments**
Hendrickson et al. [3]	102	RC vs. PI	1:1.8 vs. 1:3.6	1:6.7 vs. 1:5.9	38% vs. 23% (p=0.35)	The majority of pre-MTP (80%) and MTP (72%) patients had at least one abnormal coagulation value on presentation to the ED^1^
Chidester et al. [20]	55	PI	1:3 vs. 1:3	NR	45% vs. 45% (p value NR)	Early coagulopathy^2^ initiated MTP. 4 thrombo-embolic events in the non-MTP group vs. 0 in the MTP group
Dressler et al. [56]	1	Case	4:4	5:4	NA	
Pickett et al. [57]	1	Case	3:6	5:6	NA	
Paterson et al. [58]	1	Case	1:1.5	1:1.5	NA	

^1^Abnormal coagulation parameters defined as prothrombin time (PT) >15.9 sec, partial thromboplastin time (PTT) >42.1 sec, fibrinogen <180 mg/dl or platelets < 185 × 10^9^/l.

^2^Early coagulopathy defined by a PTT > 36 seconds.

RC: retrospective cohort; PI: prospective intervention; Case: case report; FFP: fresh frozen plasma; RBC: red blood cells; PLT: platelet concentrate; NR: not reported; NA: not applicable; MTP: massive transfusion protocol; ED: emergency department.

Chidester et al. conducted a prospective study of 55 children, 22 of them received blood transfusions according to a MTP and 33 patients were transfused at physician discretion. Massive transfusion was at this institution defined as ≥ 1 blood volume transfused within 24 hours or half of a blood volume in 12 hours. Even though the total number of blood products transfused was greater in the MTP group, the actual blood product ratio between the groups showed no statistically significant difference with a mean ratio FFP:RBC of 1:3 in both groups. Early coagulopathy (Table 1) was associated with MTP activation. No significant difference in mortality was found between the two groups (45% vs. 45%). However, they did find that the patients in the MTP group had a significantly higher ISS, and that 4 thromboembolic events occurred in the non-MTP group versus 0 in the MTP group, which the group attributed to under transfusion of the non-MTP group (Table 1) [20].

Dressler et al. described the use of a MTP in the case of a 9-year old boy with severe intraoperative bleeding (>4 liters). They applied a blood product ratio of 4:4:5 units of RBC:FFP:PLT perioperatively and did not find signs of coagulopathy postoperatively [55].

Pickett et al. described the use of a MTP consisting of 6:3:5 ratio of RBC:FFP:PLT for pre- and perioperative transfusion in a 15-year old boy with an estimated blood loss of 10 liters due to a gunshot wound to the chest. The patient did not present with or develop coagulopathy after resuscitation [56].

Paterson et al. presented the case of a 5-year old girl with severe intracranial bleeding due to an arterio-venous malformation, resulting in an estimated loss of more than 5–6 blood volumes. Their MTP, after the loss of 2 blood volumes with expected continued bleeding, consisted of RBC, FFP and PLT given in a ratio of 30:20:20 ml/kg (total 70 ml/kg) in each cycle. The patient survived and was admitted to the ICU with a normal postoperative coagulation profile [57].

### Pro-haemostatics in paediatric trauma patients

The use of adjuvant haemostatic agents has not been systemically assessed in paediatric patients.

Thrombelastometry guided infusion of fibrinogen was described to successfully secure haemostasis in a small study of nine massively bleeding children undergoing craniosynostosis as well as in a case report depicting a child with severe abdominal and pelvic injuries following blunt trauma [58,59].

The use of tranexamic acid in paediatric patients undergoing cardiac surgery was evaluated in a systemic review from 2012 including 8 studies (848 patients). Due to heterogeneous data including marked variability in dosage, the effects of tranexamic acid on postoperative morbidity and mortality could not be evaluated. Nor could potential side effects associated with the drug [60].

Recombinant factor VIIa (rFVIIa) has been a useful adjuvant to blood component therapy in achieving haemostasis in severely haemorrhaging neonatal and paediatric patients who did not have haemophilia [61,62], and case reports have described rapid correction of coagulopathy in head-injured children with rFVIIa [63]. A retrospective study including 135 children who had received rFVIIa for off-label use, showed significantly decreased blood-product administration but also associated severe thromboembolic events in children who had received rFVIIa [64].

Hence, more research is needed before these adjuvants can be utilized beyond their restricted use and their role in the treatment of bleeding paediatric trauma patients can be established.

### Colloid use in paediatric trauma patients

Haemodilution with large amounts of colloids may have negative effects on haemostasis [65,66]. Comparing the effects of hydroxyethyl starch (HES) and human albumin (HA) on coagulation by thrombelastography in children weighing 3–15 kg, Haas et al. found significantly more impaired coagulation after the use of HES [66]. Volume replacement with HA may be associated with increased mortality in trauma patients with TBI when compared to saline [67]. Caution is therefore warranted, and colloids should not be used for volume replacement in the trauma setting given the high incidence of early coagulopathy and TBI in the paediatric trauma population [3,6,7].

### Coagulation monitoring

Coagulopathy in trauma has historically been described as multifactorial, attributed to acidemia and hypothermia combined with dilution and consumption of clotting factors and platelets secondary to fluid administration and bleeding [14]. An early acute traumatic coagulopathy induced by trauma and hypoperfusion (shock) was recently identified, as described above.

Early monitoring of coagulation is essential to identify coagulopathy, and this is routinely based on conventional plasma based coagulation tests such as PT, APTT, INR, fibrinogen and platelet count [68]. These plasma-based tests, however, only reflect the initiation of the haemostatic process and poorly correlate with clinically relevant coagulopathies [69,70]. Moreover, several of them are imprecise in young children.

Viscoelastic haemostatic assays (VHA) such as Thrombelastography (TEG®)/thromboelastometry (ROTEM®) analyse the viscoelastic properties of whole blood, thereby reflecting the entire haemostatic process allowing for a more qualitative analysis of the individual cellular components and their interactions in haemostasis [70,71]. Inherent limitations to the interpretation of VHA results are, that the inhibitory effect of antithrombotic medications such as clopidogrel and aspirin on platelet aggregation can only be assessed using the TEG® Platelet Mapping assay. This assay has been used perioperatively to evaluate the activation of arachidonic acid and adenosine diphosphate pathways, specifically assessing the potential inhibition of those pathways by aspirin and clopidogrel [72,73].

The use of VHA in massively bleeding paediatric patients have not yet been firmly established.

Vogel et al. recently found that rapid TEG (rTEG) on admission was a useful real-time tool in assessing coagulopathy and directing haemostatic resuscitation in paediatric trauma patients. Furthermore, the admission rTEG was predictive of early transfusion requirements and correlated with outcome [74]. These results are in alignment with other investigators including our own group, where TEG® has been shown to reflect acute coagulopathy of trauma and predict morbidity and mortality in adult trauma patients [75-77]. A further advantage of applying TEG®/ROTEM® compared to standard plasma based tests is that they provide faster results [33,75]. Importantly, age-specific reference values exist for the use of TEG®/ROTEM® in paediatric patients [32,78]. From data based on 359 children aged 0 months to 16 years, an attempt to establish age-dependent reference values for ROTEM® assays revealed that children aged 0–3 months, despite showing prolonged standard plasma coagulation test results (prolonged PT and APTT), exhibited accelerated coagulation times compared to older children and adults, and a strong clot firmness within the range of adults. Lysis indices of < 85% were observed in nearly one third of all children less than 16 years of age without increased bleeding tendency [32]. Chan et. al did not find age-related differences in kaolin-activated TEG variables, and attributed this to the balanced maturational differences in coagulation factor levels and coagulation inhibitors in children. However, they did conclude that reference values depend on the specific analyzers and activators used [78], since significant differences in R and K times have been found when using kaolin versus celite as an activator [79].

Early identification and implementation of goal-directed treatment of traumatic coagulopathy could, potentially, improve outcome in trauma patients. Therefore, the use of VHA to assess coagulopathy and to guide blood transfusion therapy has been recommended in the recent European guidelines regarding management of massively bleeding trauma patients [68].

**Figure 1:**
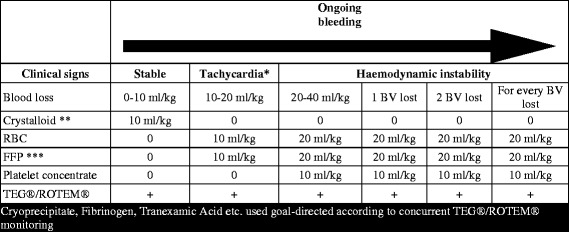
**Massive transfusion protocol for paediatric trauma patients in Copenhagen, Denmark.** BV: estimated blood volume; RBC: red blood cells; FFP: fresh frozen plasma. * Or narrowed pulse pressure or other signs of hypovolemia: ** No use of colloids (synthetic or natural): *** Thawed FFP is available ensuring early use.

At our institution, both adult and paediatric patients with uncontrolled bleeding are treated with predefined transfusion packages, based on HR principles involving early administration of FFP (pre-thawed plasma is always available for immediate delivery) and PLT (whole blood derived platelet concentrate) [80]. The transfusion package encompasses five units of RBC, five units of FFP and two units of platelet concentrates for adults whereas for children, the recommended ratio is 20:20:10 ml/kg of RBC:FFP:PLT (Figure 1). Based on the results of real-time TEG® analyses, goal-directed treatment with specific blood products and pro-coagulant adjuvants (tranexamic acid, cryoprecipitate, fibrinogen) are administered according to protocol and expert opinion, basing interventions primarily on the clinical presentation of the patient together with the real time TEG® results. The implementation of this MTP has led to increased survival of massively bleeding adult patients at our institution [15,80], but results from paediatric data are still pending.

Romlin et al. found that the routine use of intraoperative ROTEM® in paediatric cardiac surgery to guide transfusions was associated with a reduced number of patients receiving transfusions, and that fewer patients in the study group received RBC and FFP, whereas more were transfused with platelet and fibrinogen concentrates [81]. A reduction in bleeding and in the number of patients requiring transfusion of FFP and PLT with VHA monitoring was confirmed by a meta-analysis on adult patients from the Cochrane institute [82]. The ability of VHA guided HR to reduce the total number of blood products transfused is an important attribute, considering the possible adverse effects associated with transfusion therapy in critically ill children.

### Risks associated with blood product transfusion in children

Safety issues relating to the transfusion of blood products include infectious and non-infectious risks. Transmission of infectious diseases are extremely rare, but children have a higher incidence of adverse reactions to transfusion of blood products than adults - and more than 80% of these are estimated to be caused by human errors [83]. Febrile non-haemolytic transfusion reaction is the most common reaction encountered, whereas acute haemolytic transfusion reactions due to AB0 incompatibility account for the vast majority of transfusion-related deaths [21]. Moreover, transfusion of blood products can cause volume overload, electrolyte disturbances often with hypocalcemia and hyperkalemia as well as dilutional coagulopathy [21].

Pieracci et al. did a retrospective pilot study of 43 paediatric trauma patients. This group found that early RBC transfusion (<6 hours from admission) was associated with increased mortality, and that late RBC transfusion (≥6 hours from admission) was associated with an increased length of stay in the ICU and more ventilator days compared to non-transfused patients [84]. However, only 30% of the early fatalities were due to exsanguination, central nervous system injury was the main cause of death. Similarly, Stone et al. identified an association between transfusion of RBC within the first 24 hours after admission and increased mortality, prolonged duration of mechanical ventilation and ICU admission in a larger retrospective study of paediatric trauma patients [85]. However, shock index and ISS were all significantly higher in the transfused group. Since both these studies were based on retrospective data, the results are likely to be influenced by indication bias given the differences in demographics of the groups being compared. A dose-dependent relationship between the number of RBC transfusions and mortality was observed by Kneyber et al. in critically ill paediatric ICU patients but interestingly, no correlation was found between the pre-transfusion haemoglobin concentration and mortality [86].

Like that observed for RBC, plasma transfusions have also been found to be independently associated with an increased occurrence of new or progressive MOF, nosocomial infections and prolonged hospitalization of critically ill children [87].

The results outlined above are all from descriptive, retrospective studies. This entails the risk of confounding by indication and no cause-effect relationship between blood product transfusion and increased morbidity or mortality can be firmly established using the results of these descriptive studies. In the TRIPICU trial, there was no change in mortality when being restrictive with RBC and only an insignificant reduction of nosocomiel infections from 25 to 20% in the restrictive group [28]. Transfusion of RBC stored for more than 14 days has, however, shown an association with increased incidence of multiple organ dysfunction and longer intensive care unit stays in critically ill children [88,89].

Considering the outlined possible risks of blood product administration, it should be noted that Cotton et al. found that implementing a predefined MTP actually reduced the incidence of MOF, infectious complications and the number of ventilator days in severely injured adult trauma patients [9]. Patients transfused according to the MTP also had reduced mortality. Though receiving more blood products intraoperatively, patients who were transfused according to the MTP had lower total transfusion requirements within the first 24 hours compared to controls [17], and this result is in alignment with Johansson et al. [90]. These findings support the hypothesis that early and aggressive administration of FFP and PLT can improve haemostasis, reduce bleeding and subsequently improve outcome.

## Conclusions

Haemorrhage is a leading cause of death in paediatric trauma patients. Predefined MTP have the potential to significantly reduce mortality by treating haemorrhagic shock and coagulopathy, thereby ensuring adequate oxygen delivery and haemostasis in massively bleeding paediatric trauma patients, especially considering the high prevalence of early coagulopathy in this population. The triggers for MTP activation in paediatric trauma patients and the optimal blood product ratio that will increase survival in massively bleeding paediatric trauma patients still have to be determined. Despite not yet having been tested in paediatric populations, MTP with increased PLT to FFP to RBC ratios combined with VHA guided component therapy seem promising, based on results in adult patients. Prospective randomized trials investigating this therapeutic approach in paediatric trauma populations are highly warranted.
